# Maternal Readiness for Newborn Self-Care in the Early Postpartum Period: Associations with Maternal Psychophysical State and Declared Breastfeeding Readiness

**DOI:** 10.3390/jcm15124522

**Published:** 2026-06-11

**Authors:** Anna Prokopowicz, Kinga Tułacz, Kamila Drobina, Łukasz Lewandowski, Izabella Uchmanowicz

**Affiliations:** 1 Division of Fundamentals of Midwifery, Department of Midwifery, Faculty of Nursing and Midwifery, Wroclaw Medical University, 51-618 Wroclaw, Poland; kinga.tulacz@umw.edu.pl (K.T.); kamila.drobina@umw.edu.pl (K.D.); 2 Department of Biochemistry and Immunochemistry, Faculty of Medicine, Wroclaw Medical University, 50-368 Wroclaw, Poland; lukasz.lewandowski@umw.edu.pl; 3 Division of Research Methodology, Department of Nursing, Faculty of Nursing and Midwifery, Wroclaw Medical University, 51-618 Wroclaw, Poland; izabella.uchmanowicz@umw.edu.pl

**Keywords:** newborn care, rooming-in, postpartum period, breastfeeding readiness, anxiety, psychological flexibility, maternal mental health, screening

## Abstract

**Objectives:** To assess maternal readiness for newborn self-care and its associations with breastfeeding readiness and psychophysical condition in early postpartum rooming-in care. **Methods:** This cross-sectional study included 200 women at 48–72 h postpartum. Maternal readiness was assessed with three 0–10 self-report scales: daytime newborn care, nighttime newborn care, and breastfeeding readiness. Psychometric, pain, anxiety, obstetric, haemoglobin, and haematocrit data were analysed using stepwise ordinal regression with bootstrap sensitivity analyses. **Results:** Breastfeeding readiness was the strongest correlate of daytime and nighttime caregiving readiness, with a marked and partially non-linear gradient (OR ≈ 13 for linear trend, *p* < 0.001). Higher anxiety on day 2 was associated with lower readiness across all domains (daytime care: OR = 0.61; nighttime care: OR = 0.69; breastfeeding: OR = 0.73; all *p* < 0.001). Daytime readiness was associated with sleep disturbance (lower readiness; OR = 0.63, *p* = 0.006) and goal-directed behaviour despite low mood (higher readiness; OR = 1.47, *p* < 0.001). Nighttime readiness correlated with concentration under emotional strain (OR = 1.63, *p* < 0.001) and was reduced in women reporting suicidal ideation (OR = 0.24, *p* = 0.012). Breastfeeding readiness was associated with greater current engagement in breastfeeding (OR = 1.90, *p* < 0.001) and higher parity (OR = 2.46, *p* = 0.002), while sleep disturbance was associated with lower readiness (OR = 0.69, *p* = 0.013). Somatic factors and social support were not independent predictors, while psychological variables showed stronger associations with readiness. **Conclusions:** Maternal readiness for newborn self-care is related to breastfeeding readiness but remains a distinct, psychologically shaped construct. These findings question the assumption that breastfeeding readiness reflects readiness for continuous newborn care. Assessment of maternal readiness may help identify support needs and guide flexible postpartum care.

## 1. Introduction

As part of efforts to support breastfeeding, the model of postpartum care recommended by the World Health Organization (WHO) is rooming-in, defined as continuous, 24 h mother–infant contact from the moment of birth [1,2]. This model aims to strengthen mother–infant bonding and promote breastfeeding; however, empirical evidence indicates that some women do not fully adhere to it, preferring partial relief, particularly during nighttime or in situations of overload [1,3]. At the same time, factors such as fatigue, pain, and postpartum anxiety have been shown to substantially limit the actual ability to provide continuous newborn care, even in the presence of a positive attitude towards breastfeeding [4,5].

Despite the growing importance of the rooming-in model, current clinical care standards do not include routine assessment of maternal readiness for independent newborn care. In practice, it is assumed that every postpartum woman possesses sufficient resources to undertake such care. There is also a lack of simple and systematic tools to assess this readiness. In particular, the relationship between breastfeeding readiness and readiness for continuous newborn self-care in the early postpartum period remains insufficiently explored, especially in the context of real fatigue, pain, and maternal psychophysical condition during the first days after childbirth [1,3,4,5].

### 1.1. Postpartum Pain as a Limiting Factor in Maternal Functioning

Postpartum pain is one of the key factors influencing maternal functioning after childbirth. Its intensity may be modified by sociodemographic factors, including lack of partner support, which has been associated with higher perceived pain levels [6,7]. The consequences of pain extend beyond immediate discomfort—greater pain intensity has been linked to an increased risk of postpartum depression and poorer emotional functioning [8,9,10].

Additionally, postpartum pain has been shown to negatively affect mother–infant bonding and maternal self-efficacy in caregiving [11]. It is also a significant factor impairing sleep quality, increasing fatigue, and limiting the ability to engage in caregiving activities [12,13,14].

### 1.2. Blood Loss and Haematological Changes in the Postpartum Period

Childbirth is physiologically associated with blood loss, which leads to a reduction in haematological parameters such as haemoglobin concentration and haematocrit. In the postpartum period, these values are commonly reduced; however, levels of haemoglobin ≥ 11 g/dL and haematocrit ≥ 33% are generally considered within the range of acceptable adaptive changes [15,16].

More pronounced decreases in haematological parameters may result in symptoms such as fatigue, weakness, dyspnoea, and impaired cognitive functioning, which can hinder recovery and the ability to care for a newborn [17,18]. Postpartum anaemia is also associated with an increased risk of postpartum depression and difficulties in breastfeeding [16,19]. In this context, even physiological blood loss may constitute a significant burden on maternal functioning during the first days postpartum.

### 1.3. Postpartum Anxiety and Low Mood

Anxiety and low mood are among the most commonly observed psychological difficulties in the postpartum period and have a substantial impact on maternal functioning. Anxiety disorders are associated with increased parenting stress and reduced caregiving confidence [20]. Depressive symptoms, in turn, negatively affect mother–infant interactions and maternal self-efficacy [21,22]. Anxiety and depression frequently co-occur and mutually reinforce each other, potentially leading to difficulties in initiating and maintaining caregiving behaviours, including breastfeeding [23,24].

### 1.4. Psychological Flexibility as an Adaptive Resource

Psychological flexibility is increasingly recognised as a key mechanism regulating emotional and behavioural functioning. Higher levels of psychological flexibility are associated with lower levels of anxiety and depression and better adaptation to parenting demands [25,26]. Defined as the ability to maintain goal-directed behaviour despite the presence of difficult internal experiences, it may play an important role in engaging in caregiving behaviours under postpartum burden [27,28].

### 1.5. Social Support as a Protective Factor

Social support constitutes one of the key protective resources in the perinatal period. Studies indicate that higher levels of perceived support are associated with lower levels of anxiety, depression, and stress in postpartum women, as well as better psychological functioning during the first months of motherhood [29,30]. At the same time, social support has been shown to mediate the relationship between postpartum stress and anxiety symptoms; for example, by improving sleep quality and strengthening adaptive resources [31]. In the context of hospitalisation, particularly when visitation is restricted, not only the actual support received but also its subjective perception becomes crucial, as it may regulate emotional tension and enhance the sense of safety.

### 1.6. Conceptualisation of Readiness

In the present study, readiness is conceptualised as a subjective maternal disposition encompassing perceived ability, intention, and psychological availability to engage in caregiving behaviours. It is a declarative construct that does not directly reflect actual skills or behaviours but may serve as their predictor [32,33,34,35].

### 1.7. Conceptual Model of the Study

Although the rooming-in model is widely promoted as a standard of care optimising breastfeeding, its primary aim has been to support the initiation, frequency, and maintenance of breastfeeding through continuous mother–infant contact [1,2]. Within the breastfeeding-centred care framework, breastfeeding constitutes the primary goal, whereas rooming-in serves as an organisational tool designed to facilitate and stabilise this process.

The existing literature has largely assumed that readiness for breastfeeding is equivalent to readiness for continuous, 24 h newborn self-care. However, this assumption has not been empirically verified. Qualitative data indicate that women in the early postpartum period clearly differentiate between these two domains—they may report high motivation to breastfeed while not feeling ready for independent, uninterrupted caregiving [3,36]. Moreover, an increasing number of studies highlight the need for flexible care models that allow temporary relief for the mother while maintaining the intention to breastfeed [37,38].

In the present study, a behavioural regulation perspective was adopted, in which breastfeeding readiness (maternal breastfeeding readiness) is conceptualised as a motivational component (intention and goal), whereas newborn self-care readiness represents the behavioural enactment of this intention. In this framework, readiness to breastfeed may initiate and direct caregiving behaviours; however, their actual implementation depends on the mother’s available psychophysical resources.

Thus, treating breastfeeding readiness as a predictor and newborn self-care readiness as an outcome variable is theoretically, systemically, and clinically justified. This approach allows for capturing a potential gap between intention and the ability to act in the early postpartum context, characterised by fatigue, pain, and emotional burden. From this perspective, rooming-in is not a goal in itself, but rather a strategy for achieving breastfeeding-related outcomes—effective only when aligned with the mother’s actual resources and readiness.

### 1.8. Aim of the Study

The aim of this study was to assess maternal readiness for newborn self-care in the early postpartum period and its associations with breastfeeding readiness and psychophysical status, including pain, haematological changes, anxiety, mood, social support, and psychological flexibility.

The study addresses an important gap in the literature regarding the interrelationships among these variables, and provides an empirical basis for the development of a simple clinical tool for assessing maternal readiness for newborn self-care within the rooming-in model, understood as a form of care that requires alignment with the mother’s actual psychophysical resources and capacities.

### 1.9. Hypothesis

We hypothesised that maternal readiness for newborn self-care in the early postpartum period and breastfeeding readiness represent distinct, although related, constructs. We further assumed that psychological and psychophysical factors, including psychological flexibility, anxiety, depressive symptoms, pain intensity, and perceived social support, may also be associated with readiness for daytime and nighttime newborn care. These factors may modify or attenuate the association between breastfeeding readiness and newborn self-care readiness, particularly when the intensity of these factors is high.

## 2. Materials and Methods

### 2.1. Study Design and Setting

The study was conducted in a tertiary-level university hospital (University Clinical Hospital in Wrocław, Poland) in a maternity ward operating under a rooming-in model. Participants were women 48–72 h postpartum. Data were collected between December 2020 and April 2021 during the COVID-19 pandemic. Due to pandemic restrictions, a complete visitor ban was in place throughout the study period. Despite the absence of external support, the rooming-in system did not allow temporary transfer of a healthy newborn to staff care based solely on maternal request. Mothers received support from midwives and a lactation consultant directly in their rooms, where they remained with their newborns 24 h a day.

### 2.2. Participants

Eligible participants were adult women who delivered a singleton, term newborn (≥37 weeks of gestation) in good condition (Apgar score 8–10), either vaginally or by caesarean section. Inclusion required good maternal condition postpartum (mobilised and clinically assessed as suitable for transfer to the rooming-in ward).

Exclusion criteria included: severe obstetric complications preventing newborn care, COVID-19 infection, newborn Apgar score < 8, and documented or self-reported history of schizophrenia or bipolar disorder.

### 2.3. Ethical Considerations

Written informed consent was obtained from all participants. The study was approved by the Bioethics Committee of Wroclaw Medical University (No. KB–747/2020). The study was not preregistered.

### 2.4. Data Collection and Measures

After providing consent, participants completed a structured questionnaire including sociodemographic data and self-report scales. Clinical and obstetric data were obtained from maternal and neonatal medical records.

### 2.5. Psychometric Measures

Psychological inflexibility was assessed using the Acceptance and Action Questionnaire-2 (AAQ-2), with higher scores indicating greater experiential avoidance (Cronbach’s α = 0.855) [39,40].

Psychological flexibility (committed action) was measured using the Committed Action Questionnaire-8 (CAQ-8), with higher scores reflecting greater engagement in value-consistent behaviour (α = 0.823) [41,42].

Depressive symptoms were assessed using the Patient Health Questionnaire-9 (PHQ-9), where higher scores indicate greater severity (α = 0.838) [43,44].

Perceived social support was measured using the Multidimensional Scale of Perceived Social Support (MSPSS), including Family, Friends, and Significant Other subscales (α = 0.909) [45,46].

### 2.6. State Measures

Pain and anxiety were assessed using Numeric Rating Scales (NRS and NRS-A), ranging from 0 to 10, where 0 indicated no pain/anxiety and 10 the worst imaginable pain/anxiety. For descriptive purposes, scores of 1–3 were interpreted as low intensity, 4–6 as moderate, and 7–9 as high.

### 2.7. Readiness Measures

Maternal readiness is considered a subjective adaptive capacity to engage in newborn self-care in the early postpartum period, taking into account the mother’s current psychophysical state, including pain, anxiety, and emotional resources on a given day. It was assessed using three single-item self-report scales referring to daytime newborn care, nighttime newborn care, and breastfeeding readiness assessing the maternal readness on a scale from 0 to 10:

- Newborn Self-Care Readiness during the Day (NSCR-D);
- Newborn Self-Care Readiness during the Night (NSCR-N);
- Breastfeeding Readiness (BFR).

Each scale reflected the mother’s subjective evaluation of her readiness in the current psychophysical state (48–72 h postpartum), where 0 indicated a complete absence of readiness, and 10 indicated maximal readiness.

Scores of 1–3 were interpreted as low readiness, 4–6 as moderate readiness, and 7–9 as high readiness.

### 2.8. Feeding Proportion

The proportion of breastfeeding was assessed using the Newborn Feeding Proportion (NFP) scale (1–5), ranging from exclusive formula feeding to exclusive breastfeeding. This approach is consistent with previously applied simplified categorical measures of infant feeding practices [47,48].

### 2.9. Haematological Measures

Haemoglobin (Hb) and haematocrit (Hct) levels were obtained from routine clinical measurements. The first measurement was performed before delivery, and the second at 48–72 h postpartum. The decrease in Hb and Hct was calculated as the difference between prepartum and postpartum values, with higher values indicating greater blood loss-related change, consistent with standard clinical interpretation of postpartum haematological adaptation.

### 2.10. Statistical Analysis

All analyses were performed in R, version 4.5.0 (R Foundation for Statistical Computing, Vienna, Austria). Ordinal regression models were fitted using the clm function from the ordinal package. Variable selection using a likelihood-ratio test stepwise procedure was implemented via proportional-odds models (polr, MASS), and final effect estimates were refitted and reported from clm as odds ratios.

Maternal readiness was assessed on postpartum day 2 using three 0–10 ratings: Newborn Self-Care Readiness during the Day (NSCR-D), Newborn Self-Care Readiness during the Night (NSCR-N), and Breastfeeding Readiness (BFR). For modelling, each rating was converted into an ordered four-level outcome to preserve the rank information while avoiding an interval-scale assumption for constructs that are not psychometrically validated, particularly the experimental breastfeeding readiness domain. For descriptive purposes, score 0 was reported as ‘no readiness’, scores 1–3 as ‘low readiness’, 4–6 as ‘medium readiness’, 7–9 as ‘high readiness’, and 10 as ‘maximal readiness’. For ordinal regression modelling, score 0 was combined with scores 1–3 because of sparse observations in the lowest category. Consequently, the modelling outcomes were analysed as four ordered categories: 0–3, 4–6, 7–9, and 10. All three outcomes were analysed as ordinal variables, and BFR was also treated as ordinal when included as a covariate in the NSCR-D and NSCR-N models, rather than as a continuous score, to remain consistent with its exploratory measurement status. When BFR was used as a covariate, it was entered via orthogonal polynomial contrasts to capture monotonic and non-linear trends across ordered categories. These contrasts were used to represent trend and curvature across ordered BFR categories; their odds ratios are not interpreted as category-to-category effects.

To explore bivariate associations between readiness outcomes and clinical, behavioural, and psychometric predictors, we used biweight midcorrelation (bicor) as implemented in the WGCNA package. Biweight midcorrelation is a robust analogue of Pearson’s correlation coefficient, which down-weights observations with large deviations from the central tendency rather than excluding them, thereby reducing sensitivity to outliers while preserving information from the full sample. This approach was chosen because several analysed variables—particularly self-rated questionnaire scores and ordinal or quasi-continuous clinical measures—exhibited skewed distributions and occasional extreme values, for which standard Pearson correlation may have yielded unstable estimates. Unlike rank-based correlations, bicor retains an interpretation analogous to Pearson’s r for linear associations, while providing improved robustness under departures from normality. Correlation coefficients are therefore reported as robust correlation coefficients ranging from −1 to +1. Corresponding *p*-values were obtained using a t-distribution approximation and are treated as exploratory, with no correction for multiple testing applied.

### 2.11. Multivariate Modelling

Candidate predictors were defined a priori and represented several conceptually distinct domains to capture psychological, social and clinical aspects of early postpartum functioning. These included:

- Psychological inflexibility (all items of the Acceptance and Action Questionnaire-2 (AAQ-2));
- Psychological flexibility (all items of the Committed Action Questionnaire-8 (CAQ-8) short form, with reverse scoring applied where required);
- Perceived social support (all items of the Multidimensional Scale of Perceived Social Support (MSPSS));
- Depressive symptom severity at the item level (all nine items of the Patient Health Questionnaire-9 (PHQ-9));
- Day 2 state measures reflecting acute physical and emotional status (Numeric Rating Scale for Pain (NRS) and Numeric Rating Scale for Anxiety (NRS-A));
- A set of clinical and obstetric characteristics.

The latter comprised categorised indicators of neonatal condition (Apgar grouping), mode of delivery, maternal education level, socioeconomic situation, family situation, number of pregnancies, number of deliveries, number of children (parity), gestational age grouping, as well as centred laboratory measures and their early postpartum changes (haemoglobin, haematocrit, and their differences between measurement points). For the breastfeeding readiness model (BFR), the proportion of breastfeeding already practiced on postpartum day 2 was also included as a centred numeric predictor. Item-level psychometric predictors (AAQ-2, CAQ-8, MSPSS, PHQ-9 items) were entered as numeric item scores (per one-point increase on the original response scale) to provide parsimonious modelling while respecting their ordinal measurement.

Three separate multivariable models were built (one for each outcome) using cumulative link ordinal regression with a logit link. Continuous predictors were centred at the sample median to improve interpretability of model intercepts and reduce collinearity, while remaining on their original scales. Candidate predictors were pre-specified to cover psychological and contextual domains (AAQ-2, CAQ-8 with required reverse-scoring for selected items, MSPSS, PHQ-9 items), day 2 state measures (pain and anxiety), and selected clinical/obstetric variables, as well as the proportion of breastfeeding already practiced on day 2 for the BFR model. To ensure model stability in the presence of sparse categories, several multi-level clinical variables were systematically collapsed into broader, clinically interpretable groupings prior to modelling (e.g., education and socioeconomic/household situation collapsed into two-level summaries; Apgar collapsed into <10 (8–9) vs. 10; gestational age grouped into ≤38, 39–40, and ≥41 weeks). In addition, factor levels with very small counts were merged into an “OTHER” category using a pre-specified minimum cell size threshold (*n* < 5), and predictors with near-zero variance or non-informative single-level factors were excluded before model fitting. These procedures were implemented uniformly across outcomes to reduce instability from sparse cells and separation. The breastfeeding proportion (1–5 ordered scale) was entered as a centred numeric score to model a linear trend across categories; reported effects reflect a one-category increase on this scale.

A pre-defined modelling policy was applied to the readiness domains to avoid reciprocal adjustment between highly related outcomes measured at the same time point. Specifically, NSCR-D and NSCR-N were never considered as candidate predictors for each other. Breastfeeding readiness (BFR) was allowed as an ordinal covariate in both caregiving models (NSCR-D and NSCR-N) to account for shared readiness variance across domains; because BFR entered both caregiving models, an additional dedicated model with BFR as the outcome was fitted to characterise its association structure. In the BFR outcome model, neither NSCR-D nor NSCR-N was included as a candidate predictor.

Variable selection was treated as a dimensionality reduction tool rather than confirmatory inference and was applied systematically in each outcome using two complementary stepwise procedures: (I) an information-criterion approach based on AIC in ordinal regression (clm), and (II) a likelihood-ratio test-based stepwise procedure implemented with polr. For each outcome, results were summarised for the AIC-selected model, the *p*-value-selected model, and a pre-defined conservative final model (pre-specified a priori) comprising the intersection of predictors retained by both selection strategies (AIC ∩ *p*-value feature selection). This intersection approach was used to focus reporting on predictors that were consistently supported across selection criteria.

Sensitivity analyses focused on the robustness of the selection process and coefficient direction. Nonparametric bootstrap resampling (100 replicates) was performed separately for each outcome and each selection strategy. Sparse category handling (pre-defined collapses and the “OTHER” rule) was specified a priori and applied unchanged within each bootstrap resample. For each candidate predictor, selection frequency was calculated as the proportion of bootstrap samples in which the predictor entered the selected model. In addition, for each estimated coefficient (excluding ordinal cut-points), directional stability was quantified as the proportion of bootstrap estimates sharing the dominant sign (positive or negative), and the bootstrap distribution of coefficients was summarised using the median and selected quantiles. All findings are interpreted as multivariable associations within an ordinal modelling framework and not as causal effects or predictive performance claims.

## 3. Results

### 3.1. Characteristics of the Population Sample

The study included 200 postpartum women (Table 1). Two records showed missing PHQ-9 data; other variables showed fully complete records. The sample was predominantly socioeconomically stable and socially embedded, with most participants reporting higher education (83%), a good socioeconomic situation (87.5%), and being married (78%). Only a small minority reported poor economic conditions (1%) or living alone (5.5%), indicating that the cohort largely represented women with relatively favourable social circumstances.

Obstetrically, the group was balanced with respect to parity: 49.5% were first-time mothers and 50.5% had previous children. Most women had experienced one or two pregnancies (74%) and one or two deliveries (93.5%), while higher gravidity or parity levels were uncommon. Delivery mode was split between vaginal birth (44%) and caesarean section (56%), reflecting a mixed obstetric profile.

Neonatal condition at birth was generally good. According to Apgar classification, 91% of newborns scored 10 points, with only 9% scoring 8 or 9, suggesting a largely uncomplicated early neonatal status. Gestational age ranged from 37 to 42 weeks, with the majority of deliveries occurring at 38–40 weeks (79%), consistent with term births.

The median maternal age was 32 years [IQR 29–34], spanning from 21 to 43 years. Laboratory parameters reflected expected early postpartum physiological changes. Median haemoglobin decreased from 12.8 g/dL before birth to 11.3 g/dL on day 2, and haematocrit from 37.2% to 33.8%, consistent with peripartum blood loss and haemodilution.

With respect to acute postpartum state on day 2, women reported moderate pain intensity (median 4 on a 0–10 scale) and moderate anxiety levels (median 3/10). Self-rated readiness measures showed relatively high perceived competence: median readiness scores for Newborn Self-Care Readiness during the Day (NSCR-D), Newborn Self-Care Readiness during the Night (NSCR-N), and Breastfeeding Readiness (BFR) were 8/10.

Newborn Feeding Proportion (NFP) was already well established in many participants: the median category for the proportion of breastfeeding was 4/5, corresponding to predominantly breastfeeding, with a substantial proportion reporting exclusive breastfeeding.

Psychometrically, the sample demonstrated moderate levels of psychological inflexibility (AAQ-2 median 16), moderate psychological flexibility (CAQ-8 median 33), and mild-to-moderate depressive symptom burden (PHQ-9 median 7.5). Perceived social support was high, with MSPSS total scores clustering near the upper end of the scale (median 80), and similarly elevated subscale scores for support from a significant person, friends, and family, indicating a strongly supported social context.

Overall, the cohort represents a term postpartum population with generally favourable neonatal outcomes, relatively stable social conditions, high perceived social support, and moderate variability in psychological and affective state, providing a context in which differences in perceived caregiving and breastfeeding readiness likely reflect individual psychological and experiential factors rather than severe medical or social adversity.

### 3.2. Exploratory Robust Correlations with the Defined Outcomes

**Figure 1:**
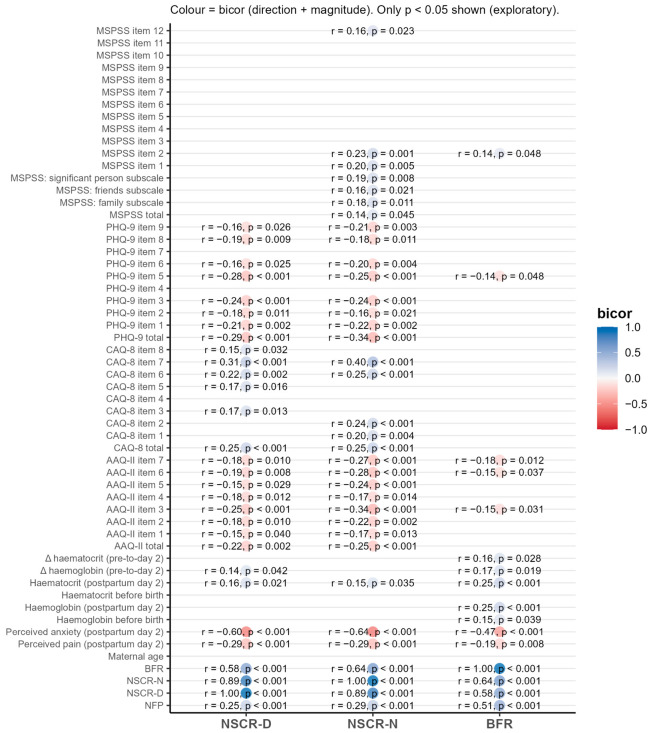
Exploratory robust correlations (biweight midcorrelation, bicor) between readiness outcomes and selected clinical and psychometric predictors.

Exploratory robust correlation analysis (Figure 1) revealed coherent patterns of association between self-rated readiness outcomes and selected clinical and psychometric variables. Readiness to care for the newborn during the day and night (NSCR-D and NSCR-N) showed strong positive intercorrelations and were also positively associated with Breastfeeding Readiness (BFR), indicating substantial conceptual overlap between these self-rated domains.

Across outcomes, higher perceived anxiety and higher perceived pain on postpartum day 2 were consistently associated with lower readiness scores. Similarly, greater depressive symptom severity (PHQ-9 total and individual items) and higher psychological inflexibility (AAQ-2) were negatively correlated with readiness outcomes, whereas psychological flexibility (CAQ-8) and perceived social support (MSPSS, particularly subscale scores) tended to show positive associations, most prominently with nighttime readiness (NSCR-N).

Clinical variables demonstrated weaker and more heterogeneous correlations. Changes in haemoglobin and haematocrit from before birth to postpartum day 2 showed small but statistically detectable associations with readiness outcomes, while maternal age and absolute haemoglobin/haematocrit values exhibited limited correlations. Newborn Feeding Proportion on postpartum day 2 (NFP) was positively associated with all readiness outcomes, most strongly with Breastfeeding Readiness.

These bivariate associations were treated as exploratory and served to inform the subsequent multivariable modelling strategy rather than to support standalone causal interpretations.

## 4. Results from Multivariate Modelling

### 4.1. Newborn Self-Care Readiness During the Day

In multivariable ordinal regression, Newborn Self-Care Readiness during the Day (NSCR-D) showed a structured pattern of associations linking breastfeeding-related readiness, current affective state, sleep-related functioning, and the ability to maintain goal-directed behaviour under emotional strain. Results of the multivariable models are shown in Table 2, and bootstrap-based sensitivity analysis in Table 3.

The strongest gradient in NSCR-D was observed across ordered categories of breastfeeding readiness (BFR). The linear component indicated markedly higher odds of being in a higher daytime readiness category with increasing breastfeeding readiness (OR = 13.00, 95% CI 5.61–30.10, *p* < 0.001), while the quadratic component suggested additional non-linearity in this relationship (OR = 3.08, 95% CI 1.57–6.05, *p* = 0.001). The cubic component was not supported (*p* = 0.640), indicating no evidence for higher-order curvature beyond the quadratic term.

Maternal anxiety on postpartum day 2 was associated with lower odds of higher daytime readiness (OR = 0.61, 95% CI 0.52–0.72, *p* < 0.001), indicating that higher acute anxiety levels co-occurred with lower perceived caregiving readiness.

At the symptom level, greater sleep disturbance (PHQ-9 item 3) was associated with lower readiness (OR = 0.63, 95% CI 0.46–0.88, *p* = 0.006), linking early postpartum sleep problems with reduced perceived daytime caregiving capacity.

Greater ability to maintain responsibilities despite feeling discouraged or low in mood (reverse-scored CAQ-8 item 6) was associated with higher daytime readiness (OR = 1.47, 95% CI 1.18–1.84, *p* < 0.001), indicating that preserved goal-directed functioning under emotional strain was aligned with higher perceived readiness.

Bootstrap sensitivity analysis showed very high selection and directional stability for breastfeeding readiness (BFR) and maternal anxiety, and moderate selection frequency but fully stable effect direction for the CAQ-8 and sleep-related predictors. These analyses characterise the structural robustness of the model rather than provide additional hypothesis testing.

### 4.2. Newborn Self-Care Readiness During the Night

In multivariable ordinal regression, Newborn Self-Care Readiness during the Night (NSCR-N) was associated with breastfeeding-related readiness, current anxiety, the ability to maintain cognitive focus under emotional strain, and a specific depressive symptom reflecting suicidal ideation. Results of the multivariable models are presented in Table 4, and bootstrap-based sensitivity analysis in Table 5.

A strong gradient in nighttime readiness was observed across ordered categories of breastfeeding readiness (BFR). The linear component showed markedly higher odds of being in a higher nighttime readiness category with increasing breastfeeding readiness (OR = 13.07, 95% CI 5.70–30.01, *p* < 0.001). The quadratic component was also supported (OR = 2.14, 95% CI 1.13–4.08, *p* = 0.020), indicating additional non-linearity in this association. The cubic component was not supported (*p* = 0.724), suggesting no evidence for higher-order curvature beyond the quadratic trend.

Maternal anxiety (NRS-A) on postpartum day 2 was associated with lower nighttime readiness (OR = 0.69, 95% CI 0.59–0.79, *p* < 0.001), indicating that higher acute anxiety levels co-occurred with lower perceived readiness to cope with nighttime caregiving.

An additional component concerned the ability to stay focused despite worry and emotional strain. Women scoring higher on the reversed CAQ-8 item 7 (greater ability to maintain concentration under emotional load) had higher odds of greater nighttime readiness (OR = 1.63, 95% CI 1.27–2.08, *p* < 0.001), linking preserved task-focused functioning with perceived capacity for nighttime care.

At the symptom level, greater endorsement of suicidal ideation (PHQ-9 item 9) was associated with lower nighttime readiness (OR = 0.24, 95% CI 0.08–0.73, *p* = 0.012), indicating that this specific severe depressive symptom co-occurred with substantially reduced perceived readiness.

Bootstrap sensitivity analysis showed near-complete selection and directional stability for breastfeeding readiness (linear and quadratic components) and maternal anxiety. The CAQ-based focus variable and suicidal ideation showed moderate selection frequency but very high directional stability, indicating that when these predictors entered resampled models, their effect direction was highly consistent. These analyses characterise structural robustness rather than provide additional hypothesis testing.

### 4.3. Breastfeeding Readiness

In multivariable ordinal regression, perceived Breastfeeding Readiness (BFR) was associated with current affective state, actual feeding behaviour, parity, and selected symptom-level psychological characteristics. Results of the multivariable models are presented in Table 6, and bootstrap-based sensitivity analysis in Table 7.

Maternal anxiety on postpartum day 2 was associated with lower odds of higher breastfeeding readiness (OR = 0.73, 95% CI 0.65–0.83, *p* < 0.001), indicating that higher acute anxiety co-occurred with lower perceived readiness for breastfeeding.

A clear behavioural gradient was observed for the proportion of breastfeeding already practiced on day 2. Greater engagement in breastfeeding (vs. other feeding methods) was associated with higher readiness categories (OR = 1.90, 95% CI 1.50–2.39, *p* < 0.001), linking current feeding practice with perceived readiness for breastfeeding.

Parity was also associated with higher readiness: women with two children had higher odds of being in a higher readiness category than women with one child (OR = 2.46, 95% CI 1.38–4.38, *p* = 0.002).

At the symptom level, sleep disturbance (PHQ-9 item 3) was associated with lower readiness (OR = 0.69, 95% CI 0.52–0.92, *p* = 0.013). In contrast, greater difficulty concentrating (PHQ-9 item 7) was associated with higher readiness categories (OR = 1.39, 95% CI 1.01–1.92, *p* = 0.045). These associations reflect multivariable co-occurrence patterns and should not be interpreted as direct beneficial or harmful effects of specific symptoms.

Bootstrap sensitivity analysis indicated very high directional stability for all predictors retained in the final model. Maternal anxiety and Newborn Feeding Proportion already practiced showed near-complete selection frequency and sign stability. Parity and the two symptom-level variables showed moderate selection frequency but consistently stable effect direction across resamples. These analyses were intended to characterise structural robustness of the model rather than to provide additional hypothesis testing.

## 5. Discussion

The aim of the present study was to assess maternal readiness for newborn self-care in the early postpartum period in relation to breastfeeding readiness and psychophysical status. A clinically relevant finding was the identification of a subgroup of women reporting zero or low readiness to care for their newborn. This indicates that even within the standard rooming-in model, there are patients who do not feel prepared to undertake caregiving in the first days after birth, highlighting the need for early identification and targeted support [49].

Readiness for newborn care, both during the day and at night, was strongly associated with breastfeeding readiness, although this relationship showed partial non-linearity. Despite a high correlation between daytime and nighttime readiness, the models differed in predictor structure. In both cases, higher anxiety on postpartum day 2 was associated with lower readiness, underscoring the central role of current emotional state in shaping perceived caregiving competence.

Differences between daytime and nighttime readiness suggest distinct functional contexts. Daytime readiness was linked to sleep quality and the ability to maintain activity despite low mood, indicating a stronger role of general physical and behavioural functioning. In contrast, nighttime readiness was more closely related to the ability to sustain attention under emotional strain and was reduced in the presence of depressive symptoms. These findings are consistent with evidence indicating that sleep quality and psychosocial resources mediate postpartum anxiety and maternal functioning [31], while sleep disturbance and fatigue are recognised risk factors for depressive symptoms in the postpartum period [50]. Together, this suggests that insufficient recovery and psychological burden may significantly impair caregiving capacity, particularly at night.

Breastfeeding readiness on postpartum day 2 was associated with current affective state, behavioural engagement, and maternal experience. Higher anxiety significantly reduced breastfeeding readiness, emphasising the importance of acute emotional state. Parity was also a differentiating factor, with multiparous women demonstrating higher readiness than primiparous women, likely reflecting prior experience and greater confidence. These findings align with previous studies showing that maternal experience supports breastfeeding self-efficacy and adaptation to the parental role [51,52,53]. Qualitative data further highlight the multidimensional nature of breastfeeding readiness, including motivation, support systems, experience, and access to resources [54].

Haematological parameters, pain, and perceived social support were not retained in the final multivariable models, likely reflecting the relatively physiological postpartum course in this cohort and the specific organisational context of the COVID-19 pandemic, which limited direct social support [55,56]. However, correlation analyses suggested potential indirect associations, indicating that these factors may still contribute to readiness through more complex pathways.

Given the lack of prior studies using analogous measures of NSCR-D/N and BFR, interpretation is based on related constructs such as maternal self-efficacy and adaptation to motherhood. In previous studies, maternal self-efficacy has consistently been identified as a protective factor associated with lower stress and depressive symptoms and better adaptation [57,58].

Previous research on newborn care readiness has focused on narrower operational aspects, such as hygienic care [59,60], and demonstrated associations with maternal experience, education, and lifestyle factors [61]. System-level factors also play a role, with higher satisfaction with prenatal care linked to greater breastfeeding self-efficacy and readiness [62]. In contrast, readiness is more commonly studied as readiness for hospital discharge, which, although related, does not fully capture immediate caregiving capacity in the early postpartum period [63,64].

Intervention studies suggest that targeted postpartum education and support can simultaneously improve caregiving readiness and breastfeeding outcomes [65], indicating that these domains are part of a shared adaptation process.

Importantly, the present study introduces a broader conceptualisation of readiness, capturing a subjective, global perception of preparedness for caregiving across both daytime and nighttime contexts. This approach allows for the identification of individual support needs and may serve as a basis for personalised care planning.

A particularly noteworthy finding concerns the context-dependent role of social support. Nighttime readiness was positively associated with perceived support from a significant other and the ability to share emotions. This suggests that the regulatory function of social support becomes especially relevant under conditions of increased burden, such as nighttime caregiving. In the hospital context, where direct support is limited, the subjective perception of available support—even remote—may reduce emotional tension and enhance perceived safety. This is consistent with findings that social support facilitates emotional regulation and goal-directed functioning under stress [66], and buffers the impact of anxiety [67]. The absence of a similar effect during the day may reflect greater availability of professional support and more predictable caregiving conditions [68].

Overall, the findings highlight the dominant role of psychological factors in shaping caregiving readiness. Anxiety consistently emerged as a key negative correlate, in line with evidence linking it to lower perceived competence and adaptation difficulties [69]. This association may reflect not only transient postpartum distress but also broader perinatal affective vulnerability. Previous research has suggested that trait anxiety, understood as a relatively stable dimension of anxiety proneness, is associated with insecurity, emotional liability, cognitive impairment, and depressive symptoms in the perinatal period [70]. At the same time, psychological flexibility—particularly the ability to act despite distress—may be particularly relevant [26,71,72].

Somatic factors showed weaker associations. Pain was negatively correlated with readiness, but its association was weaker than that observed for anxiety, consistent with previous findings [5,73]. Notably, Shebelsky et al. [74] demonstrated that psychological factors were more important for mother–infant bonding than pain, and even severe pain did not significantly disrupt bonding. Similarly, haematological changes likely reflected physiological postpartum adaptation [75,76]. Evidence also suggests that even in cases of postpartum haemorrhage, exclusive breastfeeding can be maintained, indicating that behavioural and psychosocial factors—particularly perceived breastfeeding self-efficacy—may outweigh physiological constraints [77].

Strong evidence supports the role of breastfeeding self-efficacy in predicting actual feeding behaviours. Higher early postpartum self-efficacy predicts continued and exclusive breastfeeding [78,79]. In the present study, the proportion of breastfeeding was associated with breastfeeding readiness, suggesting that successful early feeding experiences may reinforce perceived competence and readiness.

Evidence on maternal readiness for newborn self-care in relation to breastfeeding readiness remains limited. The meta-analysis by Guo et al. [80] focused on educational interventions and showed that such programmes may indirectly reduce anxiety, which was also assessed in the present study. Other studies have examined related factors, including anxiety and social support [62,64,81]. However, we did not identify studies evaluating psychological flexibility in relation to readiness for daytime newborn care, nighttime newborn care, and breastfeeding readiness.

### 5.1. Clinical Implications

The findings have direct clinical relevance for postpartum care organisation. First, routine assessment of maternal readiness—particularly using brief self-report measures—may help identify women at risk of reduced caregiving capacity. Second, the strong role of anxiety suggests that early psychological screening and support may be critical for maintaining caregiving readiness. Third, the context-dependent importance of social support highlights the need to consider flexible care models, especially during nighttime, including the possibility of temporary relief or structured support in rooming-in settings.

### 5.2. Strengths and Limitations

A major strength of this study is the integration of psychological, clinical, and behavioural variables within a single multivariable framework, allowing for a comprehensive assessment of early postpartum functioning. The use of robust statistical methods, including ordinal modelling and bootstrap-based sensitivity analyses, further supports the stability of the findings.

However, several limitations should be acknowledged. First, this single-centre study was conducted in a tertiary hospital and included a relatively socioeconomically advantaged cohort with high perceived support, which may limit generalisability and underestimate the impact of social adversity on maternal readiness [82,83]. Second, data were collected during the COVID-19 pandemic, when restrictions on partner presence, visitation, and informal support may have influenced maternal security, anxiety, bonding, and perceived readiness [84]. Third, the cross-sectional design precludes causal inference because exposures and outcomes were assessed at the same time point [82,85]. Finally, readiness was measured using self-report scales and was not verified by objective behavioural assessment; therefore, the findings reflect perceived readiness rather than observed caregiving competence or actual newborn care behaviour [86]. Finally, these findings should also be interpreted in light of possible social desirability bias. Postpartum mothers may feel implicit pressure to report high readiness for caregiving and breastfeeding, particularly in hospital environments strongly oriented toward rooming-in practices. Therefore, declared readiness may partly reflect perceived expectations regarding “good motherhood” rather than actual caregiving capacity. In this context, anxiety may be especially relevant, as women with higher anxiety may be more likely to report lower confidence, reduced perceived competence, or greater difficulty in meeting continuous caregiving demands [70].

### 5.3. Future Directions

Future research should validate the readiness construct longitudinally and examine its predictive value for actual caregiving behaviours, breastfeeding outcomes, and maternal well-being after discharge. It would also be valuable to explore the applicability of the model in more diverse populations and different healthcare settings. Further development of a brief, clinically applicable screening tool based on these findings may support implementation in routine care. It is worth noting that the tools used consider validation and use in prospective studies, which cover the state and transition from pregnancy to the postpartum period. We therefore interpret the findings as preliminary and exploratory.

## 6. Conclusions

Our hypothesis regarding maternal readiness for newborn self-care in the early postpartum period and breastfeeding readiness was corroborated by the findings. In the present study, we found that maternal readiness for newborn self-care is strongly associated with breastfeeding readiness, supporting their interrelationship. However, distinct psychological correlates evaluated in this study indicate that they are not identical constructs. Psychological factors—particularly anxiety and psychological flexibility—may be particularly relevant, while somatic factors are of secondary importance. The findings support the potential utility of a brief readiness assessment tool as a low-burden screening instrument in clinical practice, enabling more individualised and responsive postpartum care.

## Figures and Tables

**Table 1:** Characteristics of the population sample.

Sociodemographic, Obstetric, and Early Postpartum Clinical Variables		
Variable	Category/Description	Values
Maternal education	Primary	1 (0.5)
	Lower secondary	3 (1.5)
	Vocational	11 (5.5)
	Secondary	19 (9.5)
	Higher education	166 (83.0)
Socioeconomic situation	Poor	2 (1.0)
	Average	23 (11.5)
	Good	175 (87.5)
Family situation	Single	11 (5.5)
	In a relationship	33 (16.5)
	Married	156 (78.0)
Mode of delivery	Vaginal	88 (44.0)
	Caesarean section	112 (56.0)
Parity (number of children)	First child	99 (49.5)
	Subsequent child	101 (50.5)
Number of pregnancies	1	85 (42.5)
	2	63 (31.5)
	3	31 (15.5)
	4	12 (6.0)
	5	8 (4.0)
	7	1 (0.5)
Number of deliveries	1	100 (50.0)
	2	87 (43.5)
	3	10 (5.0)
	4	2 (1.0)
	5	1 (0.5)
Neonatal condition (Apgar)	8 points	10 (5.0)
	9 points	8 (4.0)
	10 points	182 (91.0)
Gestational age (weeks)	37	16 (8.0)
	38	60 (30.0)
	39	62 (31.0)
	40	36 (18.0)
	41	25 (12.5)
	42	1 (0.5)
Maternal age (years)	Continuous	32 [29–34] (21–43)
Haemoglobin, before birth (g/dL)	Continuous	12.8 [12.0–13.4] (10.0–15.5)
Haematocrit, before birth (%)	Continuous	37.2 [35.7–39.2] (30.1–45.4)
Haemoglobin, postpartum day 2 (g/dL)	Continuous	11.3 [10.3–12.0] (7.8–14.0)
Haematocrit, postpartum day 2 (%)	Continuous	33.8 [31.2–35.9] (22.6–41.3)
Haemoglobin, before-to-postpartum day 2 change (g/dL)	Continuous	−1.5 [(−2.2)–(−0.8)] (−12.2–4.1)
Haematocrit, before-to-postpartum day 2 change (%)	Continuous	−3.7 [(−5.9)–(−1.9)] (−4.2–0.6)
Maternal pain intensity, postpartum day 2 (NRS 0–10)	State pain	4 [2–5] (0–9)
Maternal anxiety level, postpartum day 2 (NRS-A 0–10)	State anxiety	3 [2–5] (0–10)
Self-authored questionnaire-based information		
Variable	Category/Description	Values
Newborn Self-Care Readiness during the Day (NSCR-D)	Self-rated, 0–10	8 [7–10] (0–10)
Newborn Self-Care Readiness during the Day (NSCR-D)—original categories	No readiness, 0	1 (0.5)
	Low readiness, 1–3	6 (3.0)
	Moderate readiness, 4–6	41 (20.5)
	High readiness, 7–9	89 (44.5)
	Maximum readiness, 10	63 (31.5)
Newborn Self-Care Readiness during the Night (NSCR-N)	Self-rated, 0–10	8 [5–10] (0–10)
Newborn Self-Care Readiness during the Night (NSCR-N)—original categories	No readiness, 0	2 (1.0)
	Low readiness, 1–3	18 (9.0)
	Moderate readiness, 4–6	45 (22.5)
	High readiness, 7–9	80 (40.0)
	Maximum readiness, 10	55 (27.5)
Breastfeeding Readiness (BFR)	Self-rated, 0–10	8 [5–10] (0–10)
Breastfeeding Readiness (BFR) —original categories	No readiness, 0	9 (4.5)
	Low readiness, 1–3	21 (10.5)
	Moderate readiness, 4–6	46 (23.0)
	High readiness, 7–9	54 (27.0)
	Maximum readiness, 10	70 (35.0)
Newborn Feeding Proportion (NFP) on postpartum day 2	1 = exclusive formula feeding; 2 = predominantly formula; 3 = 50% formula/50% breastfeeding; 4 = predominantly breastfeeding; 5 = exclusive breastfeeding	4 [3–5] (1–5)
Psychometry—sums		
Variable	Description	Values
Psychological inflexibility (AAQ-2 total score)	Acceptance and Action Questionnaire-2	16 [12–21] (7–45)
Psychological flexibility (CAQ-8 total score)	Committed Action Questionnaire-8 (female version, reversed items 5–8)	33 [28–39] (11–48)
Depressive symptom severity (PHQ-9 total score)	Patient Health Questionnaire-9	7.5 [4.3–11.0] (0–26)
Perceived social support—total (MSPSS)	Multidimensional Scale of Perceived Social Support	80 [74–84] (30–84)
Support from a significant person	MSPSS subscale	28 [27–28] (12–28)
Support from friends	MSPSS subscale	27 [24–28] (4–28)
Support from family	MSPSS subscale	27 [24–28] (10–28)
Psychometry—items		
Variable	Category/Description	Values
AAQ-2 (The Acceptance and Action Questionnaire-2)	Item 1—My painful experiences and memories make it difficult for me to live a life that I would value	2 [1–3] (1–7)
	Item 2—I am afraid of my feelings	2 [1–3] (1–6)
	Item 3—I worry about not being able to control my worries and feelings	2 [1–3] (1–7)
	Item 4—My painful memories prevent me from having a fulfilling life	2 [1–2] (1–7)
	Item 5—Emotions cause problems in my life	2 [2–4] (1–7)
	Item 6—It seems like most people are handling their lives better than I am	2 [1–3] (1–7)
	Item 7—Worries get in the way of my success	2 [1–4] (1–7)
CAQ-8 (Committed Action Questionnaire-8)	Item 1—I can remain committed to my goals even when there are times that I fail to reach them	5 [4–6] (0–6)
	Item 2—When a goal is difficult to reach, I am able to take small steps to reach it	5 [4–5] (0–6)
	Item 3—I prefer to change how I approach a goal rather than quit	4 [3–5] (0–6)
	Item 4—I am able to follow my long-term plans, including times when progress is slow	4 [4–5] (1–6)
	Item 5 (reversed)—I find it difficult to carry on with an activity unless I experience that it is successful	3 [2–4] (0–6)
	Item 6 (reversed)—If I feel distressed or discouraged, I let my commitments slide	4 [3–5] (0–6)
	Item 7 (reversed)—I get so wrapped up in what I am thinking or feeling that I cannot do the things that matter to me	5 [3–6] (0–6)
	Item 8 (reversed)—If I cannot do something my way, I will not do it at all	4 [3–5] (0–6)
MSPSS (Multidimensional Scale of Perceived Social Support)	Item 1—There is a special person who is around when I am in need	7 [7–7] (3–7)
	Item 2—There is a special person with whom I can share my joys and sorrows	7 [7–7] (1–7)
	Item 3—My family really tries to help me	7 [6–7] (2–7)
	Item 4—I get the emotional help and support I need from my family	7 [6–7] (3–7)
	Item 5—I have a special person who is a real source of comfort to me	7 [7–7] (3–7)
	Item 6—My friends really try to help me	7 [6–7] (1–7)
	Item 7—I can count on my friends when things go wrong	7 [6–7] (1–7)
	Item 8—I can talk about my problems with my family	7 [6–7] (2–7)
	Item 9—I have friends with whom I can share my joys and sorrows	7 [6–7] (1–7)
	Item 10—There is a special person in my life who cares about my feelings	7 [7–7] (3–7)
	Item 11—My family is willing to help me make decisions	6 [5–7] (1–7)
	Item 12—I can talk about my problems with my friends	7 [6–7] (1–7)
PHQ-9 (Patient Health Questionnaire-9)	Item 1—Little interest or pleasure in doing things	1 [0–2] (0–3)
	Item 2—Feeling down, depressed, or hopeless	1 [0–1] (0–3)
	Item 3—Trouble falling or staying asleep, or sleeping too much	2 [1–3] (0–3)
	Item 4—Feeling tired or having little energy	2 [1–2] (0–3)
	Item 5—Poor appetite or overeating	1 [0–2] (0–3)
	Item 6—Feeling bad about yourself—or that you are a failure or have let yourself or your family down	0 [0–1] (0–3)
	Item 7—Trouble concentrating on things, such as reading the newspaper or watching television	1 [0–1] (0–3)
	Item 8—Moving or speaking so slowly that other people could have noticed? Or the opposite—being so fidgety or restless that you have been moving around a lot more than usual	0 [0–1] (0–3)
	Item 9—Thoughts that you would be better off dead or of hurting yourself in some way	0 [0–0] (0–3)

Values are reported as n (%) for categorical variables and as median [IQR] (observed min–max) for quantitative variables. Percentages are calculated within each variable, with the denominator equal to the number of non-missing observations for that variable. For quantitative variables, the median and interquartile range (IQR; 25th–75th percentile) are shown together with the observed minimum and maximum. Postpartum day numbering refers to the time since delivery (day 2 = second postpartum day). For self-rated readiness measures (NSCR), higher scores indicate greater perceived readiness on a 0–10 scale. The proportion of breastfeeding on postpartum day 2 is reported on an ordinal 1–5 scale (1 = exclusive formula feeding; 2 = predominantly formula; 3 = 50% formula/50% breastfeeding; 4 = predominantly breastfeeding; 5 = exclusive breastfeeding). For CAQ-8, items 5–8 were reverse-scored prior to calculating the total score (CAQ-8 total). For MSPSS, subscale scores are presented for support from a significant person, friends, and family; the total MSPSS score reflects the sum across all 12 items.

**Table 2:** Multivariable ordinal regression models for Newborn Self-Care Readiness during the Day (NSCR-D).

Predictor	AIC Model OR (95% CI), *p*	PV Model OR (95% CI), *p*	Final Model OR (95% CI), *p*	Interpretation
Maternal anxiety (day 2)	0.61 (0.52–0.72), *p* < 0.001	0.61 (0.52–0.72), *p* < 0.001	0.61 (0.52–0.72), *p* < 0.001	Odds ratio for being in a higher category of perceived daytime readiness to care for the baby (NSCR-D) per one-unit increase in maternal anxiety intensity on day 2 (centred at the sample median).
Breastfeeding readiness (BFR)—linear component	13.00 (5.61–30.10), *p* < 0.001	13.00 (5.61–30.10), *p* < 0.001	13.00 (5.61–30.10), *p* < 0.001	Odds ratio for being in a higher category of NSCR-D associated with the linear (monotonic) component of perceived readiness for natural breastfeeding (BFR) across its ordered categories.
Breastfeeding readiness (BFR)—quadratic component	3.08 (1.57–6.05), *p* = 0.001	3.08 (1.57–6.05), *p* = 0.001	3.08 (1.57–6.05), *p* = 0.001	Odds ratio for being in a higher category of NSCR-D associated with the quadratic (non-linear) component of the relationship between breastfeeding readiness (BFR) and daytime caregiving readiness.
Breastfeeding readiness (BFR)—cubic component	1.15 (0.64–2.08), *p* = 0.640	1.15 (0.64–2.08), *p* = 0.640	1.15 (0.64–2.08), *p* = 0.640	Odds ratio for being in a higher category of NSCR-D associated with the cubic higher-order component of the relationship between breastfeeding readiness and daytime caregiving readiness.
Maintaining responsibilities when discouraged (CAQ-8, item 6 reverse-scored)	1.47 (1.18–1.84), *p* < 0.001	1.47 (1.18–1.84), *p* < 0.001	1.47 (1.18–1.84), *p* < 0.001	Odds ratio for being in a higher category of NSCR-D per one-unit increase in the reversed CAQ-8 item 6 score, reflecting less tendency to neglect responsibilities when discouraged.
Sleep disturbance (PHQ-9 item 3)	0.63 (0.46–0.88), *p* = 0.006	0.63 (0.46–0.88), *p* = 0.006	0.63 (0.46–0.88), *p* = 0.006	Odds ratio for being in a higher category of NSCR-D per one-unit increase in PHQ-9 item 3 severity (sleep disturbance: insomnia or hypersomnia).

OR—odds ratio from cumulative link ordinal regression (logit link). All outcomes were modelled as ordered categories (no–low–medium–high–maximal readiness). Continuous predictors were centred at the sample median. AIC model: variables selected using Akaike Information Criterion. PV model: variables selected using likelihood-ratio test-based stepwise procedure. Final model: intersection of predictors retained by both selection strategies (AIC ∩ PV). Associations represent multivariable relationships and should not be interpreted as causal effects. When BFR was used as an ordinal predictor, it was represented by orthogonal polynomial contrasts (linear/quadratic/cubic) to capture monotonic and non-linear trends across ordered categories. These contrasts capture trend components across ordered categories and therefore do not correspond to a one-category increase.

**Table 3:** Bootstrap-based stability analysis of predictors of Newborn Self-Care Readiness during the Day (NSCR-D).

Predictor (Final, *p* < 0.05)	Final OR (95% CI), *p*	Selection Frequency AIC (%)	Selection Frequency PV (%)	Direction Stability AIC (%)	Direction Stability PV (%)
Maternal anxiety (day 2)	0.61 (0.52–0.72), *p* < 0.001	100	100	100	100
Breastfeeding readiness (BFR)—linear component	13.00 (5.61–30.10), *p* < 0.001	100	100	100	100
Breastfeeding readiness (BFR)—quadratic component	3.08 (1.57–6.05), *p* = 0.001	100	100	100	100
Maintaining responsibilities when discouraged (CAQ-8, item 6 reverse-scored)	1.47 (1.18–1.84), *p* < 0.001	78	66	100	100
Sleep disturbance (PHQ-9 item 3)	0.63 (0.46–0.88), *p* = 0.006	76	65	100	100

Bootstrap sensitivity analysis (B = 100 resamples) assessed the stability of the final model structure. Selection frequency indicates the proportion of bootstrap samples in which a predictor entered the selected model. Direction stability represents the proportion of bootstrap estimates with the same sign as the median coefficient. Sensitivity analysis evaluated model robustness rather than statistical significance.

**Table 4:** Multivariable ordinal regression models for Newborn Self-Care Readiness during the Night (NSCR-N).

Predictor	AIC Model OR (95% CI), *p*	PV Model OR (95% CI), *p*	Final Model OR (95% CI), *p*	Interpretation
Maternal anxiety (day 2)	0.68 (0.59–0.79), *p* < 0.001	0.67 (0.58–0.78), *p* < 0.001	0.69 (0.59–0.79), *p* < 0.001	Odds ratio for being in a higher category of perceived nighttime readiness to care for the baby (NSCR-N) per one-unit increase in maternal anxiety intensity on day 2 (centred).
Breastfeeding readiness (BFR)—linear component	12.63 (5.50–28.99), *p* < 0.001	13.58 (5.92–31.13), *p* < 0.001	13.07 (5.70–30.01), *p* < 0.001	Odds ratio for higher NSCR-N associated with the linear (monotonic) trend across ordered categories of perceived readiness for natural breastfeeding (BFR).
Breastfeeding readiness (BFR)—quadratic component	2.12 (1.11–4.06), *p* = 0.023	2.45 (1.27–4.72), *p* = 0.007	2.14 (1.13–4.08), *p* = 0.020	Odds ratio for higher NSCR-N associated with the quadratic (non-linear) component of the BFR–NSCR-N relationship.
Breastfeeding readiness (BFR)—cubic component	1.04 (0.59–1.84), *p* = 0.896	(0.57–1.77), *p* = 0.999	1.11 (0.63–1.94), *p* = 0.724	Odds ratio for higher NSCR-N associated with the cubic higher-order component of the BFR–NSCR-N relationship.
Ability to stay focused despite worry and emotional strain (CAQ-8 item 7, reversed)	1.55 (1.20–2.00), *p* < 0.001	1.64 (1.28–2.10), *p* < 0.001	1.63 (1.27–2.08), *p* < 0.001	Odds ratio for higher NSCR-N per one-unit increase in reversed CAQ-8 item 7 score, reflecting greater ability to stay focused despite worry and emotional strain.
Suicidal ideation (PHQ-9 item 9)	0.27 (0.09–0.78), *p* = 0.016	0.25 (0.07–0.90), *p* = 0.034	0.24 (0.08–0.73), *p* = 0.012	Odds ratio for being in a higher category of NSCR-N per one-unit increase in PHQ-9 item 9 severity (suicidal ideation).
Emotional sharing with a significant person (MSPSS item 2)	1.75 (1.12–2.74), *p* = 0.014	—	—	Odds ratio for higher NSCR-N per one-unit increase in MSPSS item 2 (sharing joys and sorrows with a significant person), retained only in the AIC-selected model.
Sleep disturbance (PHQ-9 item 3)	0.70 (0.52–0.96), *p* = 0.025	—	—	Odds ratio for higher NSCR-N per one-unit increase in PHQ-9 item 3 severity (sleep disturbance), retained only in the AIC-selected model.
Ability to discuss problems with friends (MSPSS item 12)	—	2.51 (1.41–4.47), *p* = 0.002	—	Odds ratio for higher NSCR-N per one-unit increase in MSPSS item 12 (ability to discuss problems with friends), retained only in the PV-selected model.
Friends as partners for emotional sharing (MSPSS item 9)	—	0.55 (0.32–0.95), *p* = 0.032	—	Odds ratio for higher NSCR-N per one-unit increase in MSPSS item 9 (friends as partners for emotional sharing), retained only in the PV-selected model.

OR—odds ratio from cumulative link ordinal regression (logit link). All outcomes were modelled as ordered categories (no–low–medium–high–maximal readiness). Continuous predictors were centred at the sample median. AIC model: variables selected using Akaike Information Criterion. PV model: variables selected using likelihood-ratio test-based stepwise procedure. Final model: intersection of predictors retained by both selection strategies (AIC ∩ PV). Associations represent multivariable relationships and should not be interpreted as causal effects. When BFR was used as an ordinal predictor, it was represented by orthogonal polynomial contrasts (linear/quadratic/cubic) to capture monotonic and non-linear trends across ordered categories. These contrasts capture trend components across ordered categories and therefore do not correspond to a one-category increase.

**Table 5:** Bootstrap-based stability analysis of Newborn Self-Care Readiness during the Night (NSCR-N).

Predictor (Final, *p* < 0.05)	Final OR (95% CI), *p*	Selection Frequency AIC (%)	Selection Frequency PV (%)	Direction Stability AIC (%)	Direction Stability PV (%)
Maternal anxiety (day 2)	0.69 (0.59–0.79), *p* < 0.001	98	100	100	100
Breastfeeding readiness (BFR)—linear component	13.07 (5.70–30.01), *p* < 0.001	100	100	100	100
Breastfeeding readiness (BFR)—quadratic component	2.14 (1.13–4.08), *p* = 0.020	100	100	98	98
Ability to stay focused despite worry and emotional strain (CAQ-8 item 7, reversed)	1.63 (1.27–2.08), *p* < 0.001	70	61	99	98
Suicidal ideation (PHQ-9 item 9)	0.24 (0.08–0.73), *p* = 0.012	71	75	100	100

Bootstrap sensitivity analysis (B = 100 resamples) assessed the stability of the final model structure. Selection frequency indicates the proportion of bootstrap samples in which a predictor entered the selected model. Direction stability represents the proportion of bootstrap estimates with the same sign as the median coefficient. Sensitivity analysis evaluated model robustness rather than statistical significance.

**Table 6:** Multivariable ordinal regression models for Breastfeeding Readiness (BFR).

Predictor	AIC Model OR (95% CI), *p*	PV Model OR (95% CI), *p*	Final Model OR (95% CI), *p*	Interpretation
Maternal anxiety (day 2)	0.73 (0.65–0.83), *p* < 0.001	0.73 (0.65–0.83), *p* < 0.001	0.73 (0.65–0.83), *p* < 0.001	Odds ratio for being in a higher category of perceived readiness for natural breastfeeding (BFR) per one-unit increase in maternal anxiety intensity on day 2 (centred at the sample median).
Proportion of breastfeeding already practiced (day 2)	1.90 (1.50–2.39), *p* < 0.001	1.90 (1.50–2.39), *p* < 0.001	1.90 (1.50–2.39), *p* < 0.001	Odds ratio for being in a higher category of breastfeeding readiness per greater proportion of breastfeeding (vs. other feeding methods) already practiced on day 2 postpartum (centred).
Parity	2.46 (1.38–4.38), *p* = 0.002	2.46 (1.38–4.38), *p* = 0.002	2.46 (1.38–4.38), *p* = 0.002	Odds ratio for being in a higher category of breastfeeding readiness for women with two children compared with women with one child (reference parity), adjusting for the other variables in the model.
Sleep disturbance (PHQ-9 item 3)	0.69 (0.52–0.92), *p* = 0.013	0.69 (0.52–0.92), *p* = 0.013	0.69 (0.52–0.92), *p* = 0.013	Odds ratio for being in a higher category of breastfeeding readiness per one-unit increase in PHQ-9 item 3 severity (sleep disturbance).
Difficulty concentrating (PHQ-9 item 7)	1.39 (1.01–1.92), *p* = 0.045	1.39 (1.01–1.92), *p* = 0.045	1.39 (1.01–1.92), *p* = 0.045	Odds ratio for being in a higher category of breastfeeding readiness per one-unit increase in PHQ-9 item 7 severity (difficulty concentrating).

OR—odds ratio from cumulative link ordinal regression (logit link). All outcomes were modelled as ordered categories (no–low–medium–high–maximal readiness). Continuous predictors were centred at the sample median. AIC model: variables selected using Akaike Information Criterion. PV model: variables selected using likelihood-ratio test-based stepwise procedure. Final model: intersection of predictors retained by both selection strategies (AIC ∩ PV). Associations represent multivariable relationships and should not be interpreted as causal effects.

**Table 7:** Bootstrap-based stability analysis of predictors of Breastfeeding Readiness (BFR).

Predictor (Final, *p* < 0.05)	Final OR (95% CI), *p*	Selection Frequency AIC (%)	Selection Frequency PV (%)	Direction Stability AIC (%)	Direction Stability PV (%)
Maternal anxiety (day 2)	0.73 (0.65–0.83), *p* < 0.001	99	99	100	100
Proportion of breastfeeding already practiced (day 2)	1.90 (1.50–2.39), *p* < 0.001	100	100	100	100
Parity	2.46 (1.38–4.38), *p* = 0.002	61	61	100	98
Sleep disturbance (PHQ-9 item 3)	0.69 (0.52–0.92), *p* = 0.013	45	47	100	100
Difficulty concentrating (PHQ-9 item 7)	1.39 (1.01–1.92), *p* = 0.045	45	62	100	100

Bootstrap sensitivity analysis (B = 100 resamples) assessed the stability of the final model structure. Selection frequency indicates the proportion of bootstrap samples in which a predictor entered the selected model. Direction stability represents the proportion of bootstrap estimates with the same sign as the median coefficient. Sensitivity analysis evaluated model robustness rather than statistical significance.

## Data Availability

The data that support the findings of this study are available from the corresponding author upon reasonable request, subject to ethical and legal restrictions pertaining to participant confidentiality.

## Abbreviations

The following abbreviations are used in this manuscript:

AAQ-2	Acceptance and Action Questionnaire-2
BFR	Breastfeeding Readiness
CAQ-8	Committed Action Questionnaire-8
CI	Confidence interval
MSPSS	Multidimensional Scale of Perceived Social Support
NFP	Newborn Feeding Proportion
NRS	Numeric Rating Scale
NSCR-D	Newborn Self-Care Readiness during the Day
NSCR-N	Newborn Self-Care Readiness during the Night
OR	Odds ratio
PHQ-9	Patient Health Questionnaire-9
WHO	World Health Organization
